# Chilean Honey as Alternative Antibacterial: In Vitro Activity Against Multidrug-Resistant Canine Bacterial Pathogens

**DOI:** 10.3390/ani16071125

**Published:** 2026-04-07

**Authors:** Mirelly Venecia Mireles-Villanueva, Jesús Humberto Reyna-Fuentes, María de la Luz Vázquez-Sauceda, María Belén Vargas, Javiera Cornejo, Mariella Neira, Ruben Alberto Muñoz-Sánchez, Lisette Lapierre

**Affiliations:** 1 Facultad de Medicina Veterinaria y Zootecnia, Universidad Autónoma de Tamaulipas, Carretera Victoria-Mante KM 5, Ejido Santa Librada, Ciudad Victoria 87274, Tamaulipas, Mexico; mirelly2508@gmail.com (M.V.M.-V.); jesushumbertoreyna@gmail.com (J.H.R.-F.);; 2 Laboratory of Food Safety, Department of Preventive Animal Medicine, Faculty of Veterinary and Animal Sciences, University of Chile, Santiago 8820808, Chile; 3 Laboratory of Veterinary Pharmacology (FARMAVET), Faculty of Veterinary and Animal Sciences, University of Chile, Santiago 8820808, Chile; 4 Department of Animal Production, Faculty of Veterinary and Animal Sciences, University of Chile, Santiago 8820808, Chile; 5 Laboratory of Bacterial Pathogens Diagnostic and Antimicrobial Resistance, Department of Preventive Animal Medicine, Faculty of Veterinary and Animal Sciences, University of Chile, Santiago 8820808, Chile

**Keywords:** minimum inhibitory concentration, honey extracts, multidrug-resistant bacteria, phenolics, flavonoids, antioxidant capacity

## Abstract

Antimicrobial resistance is an emerging challenge in veterinary medicine, particularly in dogs, where bacterial skin infections are highly prevalent. Honey and its bioactive extracts have emerged as potential natural alternatives to conventional antimicrobials. This study evaluated the antimicrobial activity of methanolic extracts from four honey types collected in Central Chile against multidrug-resistant (MDR) bacterial isolates from canine patients, including *Enterococcus faecium*, *Staphylococcus aureus*, *Escherichia coli*, *Pseudomonas aeruginosa*, *Pasteurella multocida*, and *Enterobacter cloacae*. Antimicrobial potency was assessed by minimum inhibitory concentration (MIC), and phenolics, flavonoids, and antioxidant capacity were quantified. All extracts inhibited bacterial growth, with *E. coli*, *E. faecium* and *S. aureus* being the most susceptible (MIC 3.13% *w*/*v*), while Gram-negative bacteria such a *P. aeruginosa*, *P. multocida*, and *E. cloacae* required higher concentrations (MIC 12.5% *w*/*v*). Secondary metabolite analysis revealed seasonal and apiary-related variations, with apiary 2 honey showing significantly higher phenolic (195.58 ± 4.28 mg GAE/100 g) and flavonoid (65.46 ± 4.35 mg QE/100 g) contents in summer. In contrast, antioxidant capacity (FRAP) did not differ significantly. These findings indicate that honey’s antimicrobial properties are closely related to its bioactive composition and influenced by season and floral origin, reinforcing its potential as a sustainable alternative to antibiotics in veterinary medicine under the One Health approach.

## Simple Summary

Bacterial infections are among the most common health problems in dogs, particularly those affecting the skin and urinary tract. These infections are usually treated with antibiotics; however, the growing problem of antimicrobial resistance limits therapeutic options and poses risks to both veterinary and human health. Natural alternatives, such as honey and its extracts, have recently attracted attention due to their antimicrobial, anti-inflammatory, and healing properties. This study evaluated the methanolic extracts of four types of Chilean honey against multidrug-resistant bacteria isolated from canine patients. The results showed that all honey extracts analyzed inhibited bacterial growth, with the highest activity observed against *Escherichia coli*, *Enterococcus faecium* and *Staphylococcus aureus* (minimum inhibitory concentration (MIC) 3.13% *w*/*v*). Gram-negative bacteria such as *Pseudomonas aeruginosa*, *Pasteurella multocida* and *Enterobacter cloacae* required higher concentrations for inhibition (MIC 12.5% *w*/*v*). A dose-dependent effect was observed, confirming that higher honey concentrations of extracts significantly reduced bacterial growth, with differences observed between honey types and harvest seasons. Notably, autumn honeys and specific floral origins showed higher bioactive compound content and more potent antimicrobial effects. These findings highlight the potential of honey extracts as complementary treatments or alternatives to antibiotics in veterinary medicine, contributing to more sustainable strategies within the One Health framework.

## 1. Introduction

Bacterial infections in companion animals are among the leading causes of morbidity and antimicrobial use in veterinary clinical practice, particularly in dogs, where skin and urinary tract infections are among the most frequently diagnosed conditions worldwide [1,2]. Retrospective studies conducted in veterinary hospitals have consistently identified bacterial skin infections as a major cause of consultations, with Gram-positive and Gram-negative bacteria playing a central role in their etiology [1]. In parallel, audits of antimicrobial prescriptions in veterinary teaching hospitals have demonstrated that urinary tract infections (UTIs) and skin infections are among the primary drivers of antibiotic use in dogs, highlighting their clinical and epidemiological relevance [2,3].

From a bacteriological perspective, canine skin infections are predominantly associated with Gram-positive bacteria, especially *Staphylococcus* spp., which are widely recognized as the principal etiological agents of canine pyoderma and other superficial and deep skin infections [1,4,5]. The predominance of staphylococcal species in canine dermatological infections has been consistently reported across different geographical regions and clinical settings, supporting their broader relevance in veterinary dermatology [4,5]. Conversely, UTIs in dogs are mainly caused by Gram-negative bacteria, with *E. coli* being the most frequently isolated uropathogen, followed by other enterobacterial and *Enterococcus* spp., as demonstrated in clinical studies and antimicrobial resistance analyses from veterinary hospitals and surveillance efforts in different regions [2,6,7,8,9]. This consistent epidemiological pattern supports the selection of *Staphylococcus* spp. and *E. coli* as clinically representative pathogens for bacteriological and antimicrobial evaluation.

The extensive and often empirical use of broad-spectrum antimicrobials for the treatment of canine skin infections and UTIs has significantly contributed to the emergence and dissemination of antimicrobial resistance (AMR) in companion animals [2,6,7,8]. Recent analyses have reported increasing resistance rates among both Gram-positive and Gram-negative bacterial isolates recovered from dogs, including multidrug-resistant (MDR) strains associated with recurrent infections and therapeutic failure [6,7,8]. This scenario represents a growing clinical challenge by limiting available treatment options and increasing the burden of chronic and recurrent bacterial diseases in dogs.

Beyond veterinary clinical practice, AMR in companion animals constitutes a broader public health concern. Evidence indicates that resistant bacteria and resistance determinants may be exchanged between pets and humans through close contact, reinforcing the importance of addressing AMR in dogs within a One Health framework [10,11]. Accordingly, international organizations such as the World Organization for Animal Health (WOAH) have identified AMR as a global priority and have emphasized the urgent need to reduce antimicrobial selection pressure while promoting alternative and complementary therapeutic strategies [12].

In this context, natural products have gained attention as potential alternatives or adjuncts to conventional antibiotics [4,13]. Among them, honey and honey-derived extracts exhibit multifactorial antimicrobial activity associated with their physicochemical properties and bioactive compounds, including phenolic compounds and flavonoids, which have demonstrated inhibitory effects against both Gram-positive and Gram-negative bacteria [14,15,16]. Experimental and clinical studies further indicate that honey and other bee-derived products, such as propolis, may represent promising complementary strategies for the management of skin and wound infections, particularly those involving staphylococcal infections [17,18,19].

Therefore, considering the high clinical prevalence of canine skin infections and UTIs, the well-established role of *Staphylococcus* spp. and *E. coli* as their principal etiological agents, and the increasing occurrence of multidrug-resistant (MDR) strains, the present study aimed to evaluate the antimicrobial potential of methanolic honey extracts from different honey types obtained from Central Chile against MDR bacterial isolates recovered from infected canine patients, thereby linking clinically relevant infections with targeted bacteriological evaluation.

## 2. Materials and Methods

### 2.1. Antimicrobial Activity

#### 2.1.1. Bacterial Strain

The antibacterial activity of four honeys was evaluated against six multidrug-resistant bacterial strains isolated from cutaneous infections in dogs: *E. coli*, *S. aureus*, *P. aeruginosa*, *E. faecium*, *E. cloacae*, and *P. multocida*. All strains were identified and confirmed at the Microbiology Laboratory (MICROVET), Faculty of Veterinary Medicine, Santiago de Chile, and classified as MDR based on MIC determinations using the VITEK-2 Compact system (bioMérieux, Marcy-l’Etoile, France).

Bacterial strains used in this study were obtained from the MICROVET Laboratory strain collection of clinical isolates obtained from companion animals attending veterinary clinics in the Metropolitan Region. For long-term preservation, isolates were stored at −20 °C in Tryptic Soy Broth (TSB; Oxoid Ltd., Basingstoke, UK) supplemented with 20% (*v*/*v*) glycerol. For metabolic reactivation, strains were recovered from glycerol stocks by inoculating an aliquot into TSB and incubating overnight at 37 °C under aerobic conditions. Reactivated cultures were subsequently streaked onto selective and differential solid culture media appropriate for each bacterial species. Specifically, Mannitol Salt Agar (Oxoid, UK) was used for *S. aureus*, BD Enterococcosel™ Agar (Becton, Dickinson and Company, Franklin Lakes, NJ, USA) for *E. faecium*, and MacConkey Agar No. 3 (Oxoid, UK) for Gram- negative bacteria, including *E. coli*, *E. cloacae*, and *P. aeruginosa*. Plates were incubated at 37 °C for 18–24 h under aerobic conditions. Pure cultures were obtained by subculturing representative colonies. Species identification was performed using the VITEK^®^ 2 automated system (bioMérieux, France), employing GN and GP identification cards for Gram-negative and Gram-positive isolates, respectively. Antimicrobial susceptibility testing was performed on the same platform using AST-GN98 and AST-GP80 cards (bioMérieux, France) according to the manufacturer’s instructions. Quality control procedures and instrument calibration were carried out according to the manufacturer’s recommendations. Minimum inhibitory concentration (MIC) values were interpreted as susceptible, intermediate, or resistant according to the Clinical and Laboratory Standards Institute (CLSI) guidelines applicable at the time of analysis. When available, species-specific clinical breakpoints were applied; otherwise, interpretive criteria for closely related organisms or epidemiological cutoff values were used, as recommended by CLSI.

#### 2.1.2. Honey Samples and Extraction

Four honey types were obtained from independent apiaries belonging to the University of Chile, located in the northeastern region of Chile. Although all apiaries are managed under similar institutional and environmental conditions, they are spatially separated by distances greater than 6 km and are characterized by distinct dominant local floral assemblages typical of the region. Honey samples were collected during two different harvesting seasons in 2024: summer (Types 1 and 2) and autumn (Types 3 and 4). Therefore, the differentiation among honey extracts reflects both seasonal variation and differences in predominant floral sources associated with each apiary. Upon receipt, honeys were transferred into amber glass jars and stored at 21 °C in the dark until analysis.

To determine the antimicrobial activity of the honey samples, honey component extraction was performed as described by Popova et al. [20] with modifications. To do this, 2 g of honey was dissolved in 20 mL of 70% methanol and mixed in an ultrasonic bath. The sample was centrifuged at 4500 rpm for 20 min and filtered through a 0.22 µm membrane filter (SLGSM33SS, MilliporeSigma, Merck KGaA, Darmstadt, Germany).

#### 2.1.3. Determination of Minimal Inhibitory Concentration (MIC)

Antimicrobial activity was assessed using the plate microdilution method to determine the MICs of the honeys. Assays were performed in sterile 96-well, round-bottom polystyrene microtiter plates. Inoculum preparation began with the inoculation of bacteria onto blood agar plates and incubation for 24 h. Colony suspensions were prepared by selecting 3 to 5 colonies with a sterile loop and transferring them to 3–5 mL of sterile Mueller–Hinton broth (MHB). The inoculum was adjusted to a 0.5 McFarland standard. Serial dilutions were prepared from the honey suspensions. For this study, honey dilution is expressed as a percentage (%) of a *w*/*v* ratio due to honey’s viscosity, which can cause substantial material loss during pipetting and errors [21]. Then, 10 µL of the diluted bacterial suspension was inoculated into wells containing the honey dilutions, yielding a final inoculum of 10^5^ CFU/mL.

Wells containing only broth were used as sterility medium controls, while wells containing bacteria and broth were used as positive controls. Medium inoculated with 70% methanol was used as a blank. The *E. coli* ATTC 25922 strain was used for quality control. The plates were incubated at 37 °C for 24 h. The MIC was determined to be the lowest honey concentration in the well, with the medium turbidity and growth halo remaining unchanged compared to the negative control.

The optical density was measured after 24 h of incubation at 630 nm using a spectrophotometer to assess differences in antibacterial activity among honeys.

### 2.2. Determination of Secondary Compound

#### 2.2.1. Total Flavonoids

The content of total flavonoids was determined as previously described [22]. In brief, 10 µL of the extract was mixed with 1.5 mL of 95% ethanol (Ibi Scientific, no. 15720, Dubuque, IA, USA), 2.7 µL of 10% aluminum chloride (Sigma-Aldrich, St. Louis, MO, USA), 2.7 µL of 1 M potassium acetate (Sigma-Aldrich), and 102.7 µL of distilled water, and incubated for 40 min at room temperature. The samples were read at 415 nm (iMark™ Microplate Absorbance Reader, series 11293, Bio-Rad Laboratories, Hercules, CA, USA) and quantified against a standard curve prepared with quercetin (Sigma-Aldrich, St. Louis, MO, USA). The concentration of total flavonoids is expressed as mg quercetin equivalents per g of dry matter (mg/QE/100 g^−1^).

#### 2.2.2. Total Phenols

The content of total phenols was determined using the Folin–Ciocalteu method [23]. In brief, 2 µL of the extracts was deposited into a 96-well plate and mixed with 25 µL of Folin–Ciocalteu reagent (Sigma-Aldrich). The samples were homogenized and incubated at room temperature for 5 min. Then, 125 µL of 10 mM NaCO_3_ (Sigma-Aldrich) and 49 µL of distilled water were added, and the mixture was incubated at room temperature for an additional 5 min. The samples were read at a wavelength of 750 nm and quantified using gallic acid as a standard. The concentration of total phenols is expressed as mg gallic acid equivalents per gram of dry matter (mg/GAE/100 g^−1^).

#### 2.2.3. Antioxidant Capacity by the Ferric Iron (Fe^+3^) Reduction Method (FRAP)

The FRAP method is based on the reduction of ferric iron (Fe^+3^) present in the FRAP reagent to the ferrous form (Fe^+2^) by the presence of antioxidants [24,25]. For sample reading, 900 µL of FRAP solution, 30 µL of the sample, and 120 µL of distilled water were used. The FRAP reagent was prepared by mixing 25 mL of acetate buffer (acetic acid–sodium acetate, pH 3.6), 2.5 mL of 10 mM TPTZ solution prepared in 40 mM HCl, and 2.5 mL of 20 mM FeCl_3_ solution. A blue-colored ferrous–TPTZ complex is formed, the intensity of which is proportional to the reducing capacity of the sample and can be quantified colorimetrically at 593 nm using a ferrous sulfate calibration curve. The absorbance was determined at a wavelength of 593 nm using a spectrophotometer reader model No. iMark, series 11293. For each reading, the absorbance reading of the control sample was considered. The final absorbance of the samples was compared with the standard curve of Trolox (100–1000 μmol/L) dissolved with ethanol (brand IBI SCIENTIFIC, catalog IBI 15720 at 96%).

### 2.3. Experimental Design

Antibacterial activity was evaluated using a broth microdilution assay in 96-well plates. The experimental factors included honey type (Types 1–4), harvesting season (summer vs. autumn), bacterial species (six MDR clinical isolates), and extract concentration (% *w*/*v*; serial dilutions). Each well represented one experimental unit. The following controls were included in each plate: (i) sterility control (broth only), (ii) growth control (broth + bacterial inoculum), (iii) solvent blank (broth containing 70% methanol at the same volume used in test wells), and (iv) quality control strain (*E. coli* ATCC 25922). MIC was defined as the lowest concentration showing no visible growth compared with the growth control.

### 2.4. Statistical Analysis

Data were analyzed using parametric or non-parametric approaches, depending on whether the assumptions of normality and homogeneity of variances were fulfilled. The antimicrobial activity of honey samples, expressed as minimum inhibitory concentration (MIC) and bacterial growth (optical density), was evaluated using the Kruskal–Wallis test. When significant differences were detected, Dunn’s test with Bonferroni correction or the Steel–Dwass method was applied for multiple comparisons. The relationship between honey concentration and bacterial optical density was further assessed through non-parametric correlation analyses, specifically Spearman’s rank correlation coefficient (ρ) and Kendall’s tau (τ). For secondary compounds (phenolics, flavonoids, and FRAP), one-way analysis of variance (ANOVA) was performed when assumptions were met, followed by Tukey’s test for multiple comparisons. All statistical analyses were conducted using JMP Pro 17 (SAS Institute Inc., Cary, NC, USA) [26].

## 3. Results

### 3.1. Antibacterial Activity

Six bacterial strains were isolated from exudative skin lesions of canine patients at a veterinary clinical facility. Each isolate corresponded to an independent clinical case. Bacterial identification and antimicrobial susceptibility testing were performed using the VITEK^®^ 2 Compact system (bioMérieux, France). The isolates included *Escherichia coli*, *Enterobacter cloacae*, *Enterococcus faecium*, *Pasteurella multocida*, *Staphylococcus aureus*, and *Pseudomonas aeruginosa*. The antimicrobial susceptibility profiles confirming multidrug resistance are presented in Table 1.

The antibacterial potency of the honey samples was determined in vitro according to the classification described by Albaridi et al. [27]. The MIC assay showed that the growth of the six isolates was inhibited by all four honey types (Table 2). The most susceptible pathogens were the multi-resistant strains *E. coli*, *E. faecium* and *S. aureus*, with lower MICs for all four honey types (3.13% *w*/*v*), demonstrating vigorous antibacterial activity, compared to the multi-resistant pathogens *P. aeruginosa*, *P. multocida*, and *E. cloacae*, which presented the MIC of 12.5% (*w*/*v*), demonstrating a lower antibacterial activity compared with the other strains.

**Figure 1:**
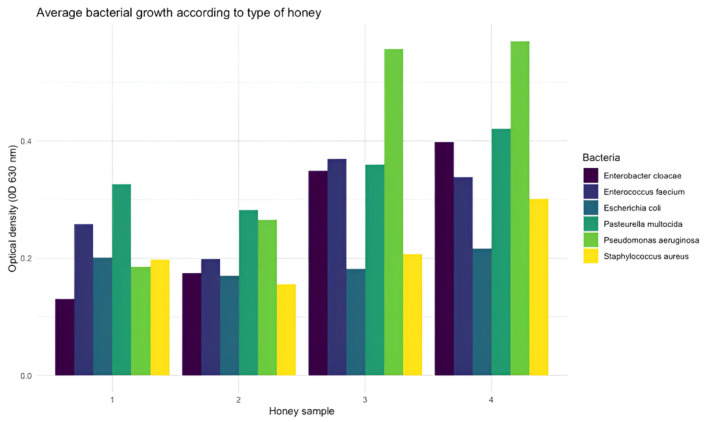
Average bacterial growth (OD630) of multidrug-resistant canine isolates exposed to four different honey types.

To assess whether there were significant differences in antimicrobial activity among the honey types analyzed, the nonparametric Kruskal–Wallis test was used, as the data did not meet the assumptions of normality and homogeneity of variance. The results showed significant differences between groups (Kruskal–Wallis, *p* < 0.05), indicating that at least one honey type exhibited antimicrobial activity different from the others (Figure 1).

Subsequently, Dunn’s multiple comparisons test with Bonferroni correction was performed to identify which groups exhibited these differences (Table 3). Although none of the pairwise comparisons reached statistical significance after the applied correction, trends close to significance were observed between type 4 honey and types 1 and 2. This suggests that type 4 honey exhibits reduced antimicrobial activity, highlighting possible variability in antimicrobial efficacy across honey types.

On the other hand, although no significant differences were obtained in the effect of the types of honey on the bacterial growth curve, it can be observed that in the presence of honey 1 and 2, the bacteria do not reach an OD ≥ 0.5, which implies a greater capacity to inhibit bacterial proliferation (Figure 1).

### 3.2. Honey Effect

The Kruskal–Wallis test revealed a highly significant effect of honey concentration on bacterial growth inhibition (χ^2^ = 189.79, df = 9, *p* < 0.0001). Median OD values decreased progressively as concentration increased, confirming a clear dose–response relationship (Table 4). Pairwise Steel–Dwass comparisons grouped concentrations into distinct categories: the lowest concentrations (0.195–0.390%) exhibited the highest median OD values, whereas intermediate concentrations (0.781–1.562%) showed a gradual reduction. Concentrations ≥ 6.25% clustered in the lowest group, indicating a plateau of maximal inhibitory effect.

**Figure 2:**
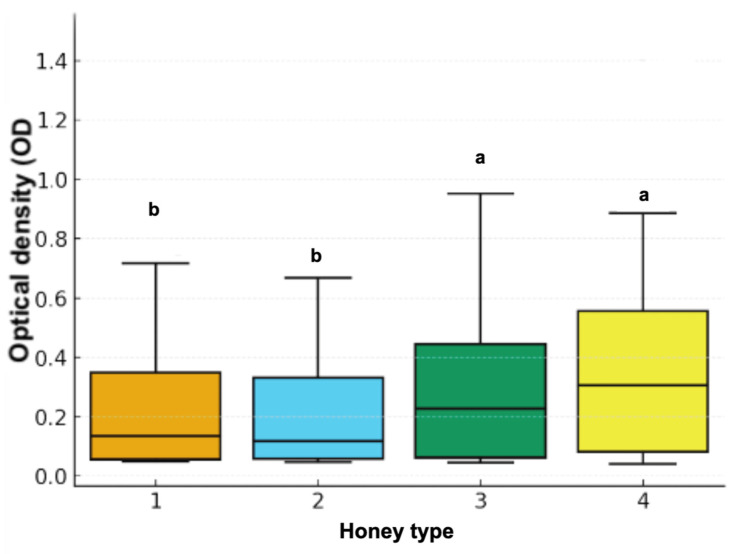
Differences in antibacterial activity among honey types based on bacterial growth inhibition measured as optical density (OD). Different letters indicate significant differences among honey types (*p* < 0.05).

**Figure 3:**
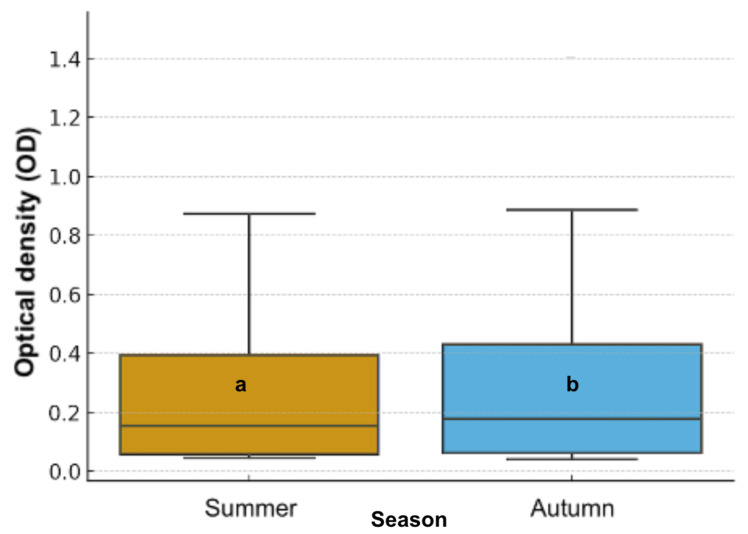
Seasonal variation in antibacterial activity of Chilean honey samples measured as optical density (OD). Different letters indicate statistically significant differences between seasons (*p* < 0.05).

Regarding honey type, significant differences were detected (χ^2^ = 9.02, df = 3, *p* = 0.029). Types 3 and 4 exhibited higher inhibitory activity, while types 1 and 2 showed lower responses (Figure 2). Seasonal comparison also showed significant variation (χ^2^ = 8.01, df = 1, *p* = 0.0046), with honeys harvested in autumn presenting greater inhibitory capacity than those collected in summer (Figure 3).

**Figure 4:**
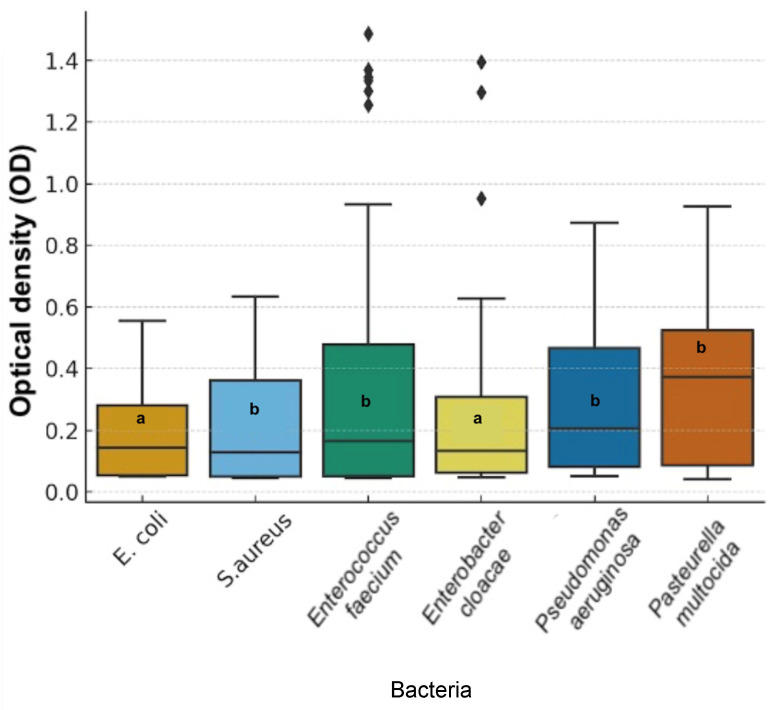
Variation in bacterial growth inhibition measured as optical density (OD_630_ nm) among bacterial species treated with honey extracts. Different letters indicate statistically significant differences among bacterial species (*p* < 0.05).

Finally, bacterial species differed significantly in their optical density (OD) to honey treatments (χ^2^ = 12.16, df = 5, *p* = 0.0327) *E. coli* and *E. cloacae* formed the most susceptible group, while *P. aeruginosa*, *P. multocida*, *S. aureus* and *E. cloacae* were the least affected (Figure 4).

**Figure 5:**
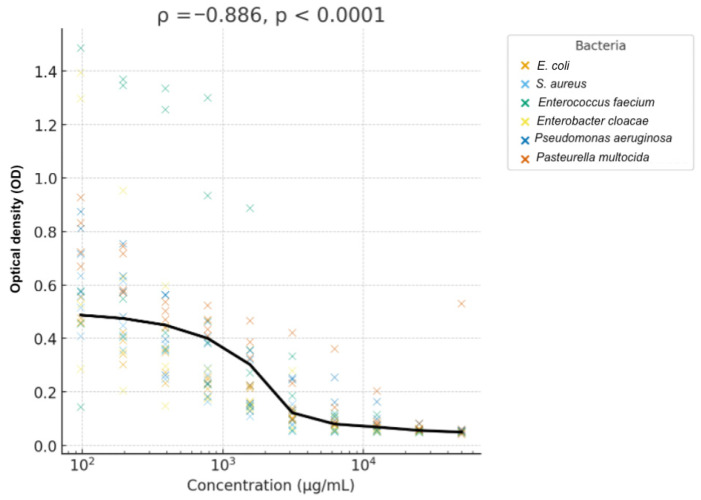
Spearman’s correlation between honey concentration (% *w*/*v*) and bacterial growth measured as optical density (OD) across bacterial species (ρ = −0.886, *p* < 0.0001).

The non-parametric correlation analysis revealed a very strong, highly significant negative relationship between honey concentration and optical density (OD). At the global level, the analysis yielded a Spearman’s ρ = −0.886 (*p* < 0.0001) and a Kendall’s τ = −0.739 (*p* < 0.0001), confirming a monotonically decreasing trend. When analyzed by bacterial species, all showed significant negative correlations with concentration. *E. coli* exhibited the strongest response (ρ = −0.981, *p* < 0.0001), indicating an almost perfect dose-dependent effect. Other species, such as *S. aureus*, also exhibited strong correlations (ρ ranging from −0.89 to −0.94), indicating a consistent pattern of bacterial growth inhibition as honey concentration increased (Figure 5).

### 3.3. Secondary Compounds

Analysis of secondary compounds in honey samples revealed significant seasonal differences. In the autumn season, no significant differences were observed in total phenolic content (*p* = 0.054) or antioxidant capacity (FRAP) (*p* > 0.05). However, flavonoid concentration differed significantly between samples (*p* = 0.024), with honey from apiary 3 (60.73 ± 0.42 mg eq/100 g) showing higher values compared to apiary 4 (39.82 ± 1.84 mg eq/100 g). In contrast, during the summer season, clear differences were detected in both phenolic and flavonoid content. Phenolic levels were significantly higher in honey from apiary 2 (195.58 ± 4.28 mg eq/100 g) compared to apiary 1 (171.49 ± 7.89 mg eq/100 g) (*p* = 0.0097). Similarly, flavonoids were significantly greater in apiary 2 (65.46 ± 4.35 mg eq/100 g) than in apiary 1 (40.66 ± 6.89 mg eq/100 g) (*p* = 0.0062). Regarding antioxidant capacity, FRAP values did not differ significantly between apiaries (*p* = 0.237), although honey from apiary 2 showed numerically higher mean values (8945 ± 1432.55 µM Trolox/100 g) than apiary 1 (7740.56 ± 452.56 µM Trolox/100 g) (Table 5).

**Figure 6:**
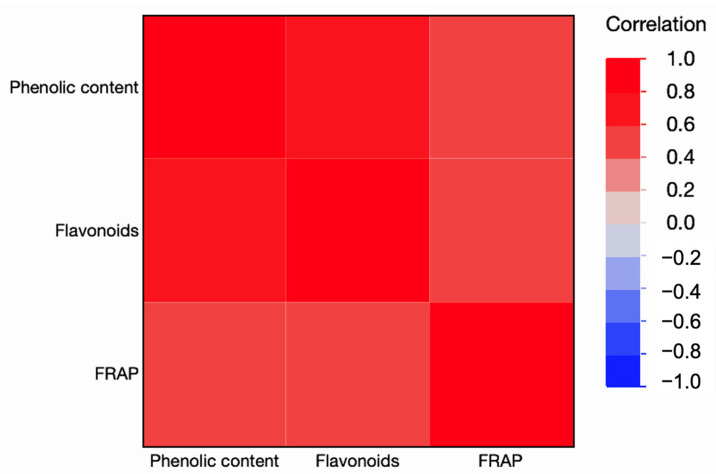
Heatmap of correlations among phenolic content, flavonoids, and antioxidant capacity (FRAP) in honey samples.

The correlation analysis among phenolic content, flavonoids, and antioxidant capacity (FRAP) revealed distinct associations between variables (Figure 6). A strong and significant positive correlation was observed between phenolic content and flavonoid concentration (r = 0.73, *p* = 0.0069), indicating that colonies with higher total phenolics also presented increased levels of flavonoids. In contrast, phenolic content showed only a moderate positive correlation with FRAP values (r = 0.55, *p* = 0.0621), which did not reach statistical significance. Similarly, flavonoid concentration was moderately correlated with FRAP (r = 0.54, *p* = 0.0686), although this association was also not significant at the 5% level. Overall, these results demonstrate a close relationship between phenolic compounds and flavonoids in honey, while the antioxidant capacity measured by FRAP exhibited only a partial association with these compounds. This suggests that, in addition to phenolics and flavonoids, other bioactive constituents of honey may contribute to its total antioxidant activity.

## 4. Discussion

The present study demonstrated that the methanolic extracts of four honey types from Central Chile exhibited antibacterial activity against multidrug-resistant bacterial isolates from canine patients. The inhibition observed across all six pathogens, particularly the low MIC values (3.13% *w*/*v*) against *E. coli*, *E. faecium* and *S. aureus*, supports previous reports indicating that honey and its bioactive components can exert strong antibacterial effects against Gram-positive and Gram-negative bacteria [15,28]. Conversely, *P. aeruginosa*, *P. multocida*, and *E. cloacae* displayed higher MIC values (12.5% *w*/*v*), consistent with their intrinsic resistance mechanisms, such as the outer membrane barrier [29]. Accordingly, pathogen selection was guided by their clinical relevance in canine skin infections and urinary tract infections, which constituted the focus of the present study [1,2]. Accordingly, these findings should be interpreted as preliminary and limited to in vitro conditions.

Interestingly, trends near significance were identified between honey type four and honeys one and two, suggesting that honey type four may have reduced antimicrobial activity. This variability among honey types may be attributed to differences in floral origin, phenolic content, and concentrations of bioactive compounds such as hydrogen peroxide and methylglyoxal, which have been shown to vary widely across geographical and botanical sources [17,30].

Although differences in bacterial growth curves were not statistically significant, the observation that bacterial optical density remained below 0.5 in the presence of honeys one and two highlights potential biological relevance. These findings suggest that certain honey types may be more effective in suppressing bacterial proliferation, even if such effects were not captured by the applied statistical corrections. Importantly, this aligns with the growing evidence that honey can act as a supportive or alternative treatment for MDR infections, potentially reducing reliance on conventional antibiotics and thereby mitigating antimicrobial resistance under the One Health approach [31,32].

The honeys analyzed showed a total phenolic content (TPC) of 169–196 mg GAE/100 g, a total flavonoid content (TFC) of 40–65 mg QE/100 g, and antioxidant capacity by FRAP of 7.6–8.9 × 10^3^ µmol TE/100 g. These values fall within the mid-to-upper range reported internationally and are comparable to several botanically defined honeys. As reviewed by [33], phenolic levels in honey vary widely with floral species, geography, and harvesting conditions, spanning from ~22.1 mg GAE/100 g in sunflower honeys [34] to >1800 mg GAE/kg in buckwheat [35] or honeydew honeys [36]. In this context, the phenolic load of our samples is comparable to that of citrus honeys (167.8 mg GAE/100 g; [37]) and *Brassica* spp. honeys (205.4–310.8 mg GAE/100 g; [38]. Similar ranges have also been reported for multifloral honeys (141–211 mg GAE/100 g; [39]. In Peru, Eucalyptus (150.08–180.37), “wild honey” (150.91–207.89), *Prosopis pallida* (121.81), and *Capparis angulata* (134.87–143.72 mg GAE/100 g) have been documented [40]. In India, multifloral honeys have been reported to contain between 74.42 and 140.83 mg GAE/100 g of total phenolic compounds. While Brassica honeys reach ~296.68 mg GAE/100 g [41]. Mexican multifloral honey has been reported at ~170 mg GAE/100 g [42]. Altogether, our values align with these benchmarks, supporting a moderate-to-high phenolic burden consistent with botanically active sources.

The flavonoid content observed in the present study (40–65 mg QE/100 g) was higher than values reported for *Acacia mangium* honeys, which typically range between 2.7 and 12.2 mg QE/100 g [33,43,44,45] and for Asteraceae honeys (3.3 mg QE/100 g) [46]. However, our values fall within the upper range reported for multifloral honeys (6.94–67.76 mg QE/100 g) [47] and remain lower than those described for certain Eucalyptus honeys, which may reach 73.5–102.1 mg QE/100 g [45]. These differences likely reflect variations in botanical origin and environmental conditions influencing flavonoid accumulation in honey. Overall, the observed TFC denotes a substantive flavonoid fraction compatible with floral sources capable of sustaining strong reducing capacity; moreover, targeted studies consistently identify quercetin, kaempferol, galangin, and related flavonoids as major contributors to antioxidant performance, modulated by geography and floral availability [48,49,50].

The evaluation of antioxidant capacity was not intended as a direct predictor of antibacterial potency but rather as a complementary functional parameter reflecting the integrated contribution of multiple bioactive constituents. The moderate correlations observed between FRAP and phenolic/flavonoid content support the concept that honey bioactivity arises from synergistic chemical interactions rather than single compounds. This aligns with the notion that, while phenolics and flavonoids are key indicators of antioxidant capacity, additional bioactive compounds (such as glucose oxidase and catalase), organic acids, minerals, and Maillard-reaction products also significantly contribute to the total reducing power [51,52]. Consistently, do Nascimento et al. [53] reported a tighter association of FRAP with TFC (r ≈ 0.66) than with TPC (r ≈ 0.36), suggesting that flavonoids, through Fe^3+^ → Fe^2+^ reduction, may play a more important role in antioxidant activity, although they are not the only contributors. Multiple studies further support strong phenolic FRAP linkages across diverse honeys, such as Brazilian honeys (highest FRAP in orange blossom; r = 0.9258) [54], Turkish honeys (r = 0.81) [55], Brazilian honeys (r = 0.8594) [49], Malaysian vs. Manuka honeys (r = 0.965; Tualang richest in phenolics) [54], and Kosovo honeys (forest honeys highest; r = 0.878) [55], whereas other datasets report weaker or non-significant associations depending on matrix and assay conditions [56]. 

Taken together, our findings place these honeys among profiles with robust antioxidant performance, and the between-apiary/season differences likely reflect genuine variation in floral supply and matrix composition. The overall evidence, particularly the closer FRAP-TFC linkage and the established roles of botany, geography, and even altitude, supports a model in which bioactivity emerges from the interaction of polyphenols (especially flavonoids) with non-phenolic cofactors, explaining why FRAP does not scale linearly with total phenolics. In addition to secondary metabolites such as phenolic compounds and flavonoids, the antimicrobial activity of honey has also been associated with its physicochemical properties, particularly its high sugar concentration and the resulting osmotic pressure, which reduces water availability and inhibits microbial growth. Honey is mainly composed of simple sugars such as fructose and glucose, which together may account for approximately 70–80% of its composition and contribute to hyperosmotic environments unfavorable for bacterial proliferation. Recent studies describe honey’s antibacterial activity as a multifactorial phenomenon involving high osmolarity, low pH, hydrogen peroxide production, and a wide range of bioactive phytochemicals that act synergistically against microorganisms [13,14,57]. However, in the present study, antimicrobial activity was evaluated using methanolic extracts, which preferentially concentrate secondary compounds and reduce the contribution of sugars in the analyzed fraction. Therefore, although osmotic effects associated with carbohydrates cannot be completely excluded, the inhibitory activity observed is likely associated with phenolic compounds and other secondary metabolites known to exert antimicrobial activity [19,57]. Nevertheless, future studies incorporating comprehensive physicochemical characterization, including sugar composition and water activity, would help further clarify the relative contribution of osmotic and phytochemical mechanisms to honey antibacterial activity.

## 5. Conclusions

This study provides a comprehensive characterization of the antimicrobial and antioxidant properties of Chilean honeys against multidrug-resistant (MDR) bacterial strains of veterinary origin. Methanolic extracts showed remarkable inhibitory activity, particularly against *E. coli*, *E. faecium* and *S. aureus*, supporting the potential of Chilean honeys as complementary agents in infection control. While variability was observed among honey types, likely due to floral and geographic differences, all samples exhibited moderate to high phenol and flavonoid content, as well as strong antioxidant capacity. Moderate correlations between the FRAP index and phenol/flavonoid levels further indicate that honey bioactivity arises from synergistic interactions among multiple bioactive compounds.

Taken together, these findings underscore the multifaceted nature of Chilean honey’s antibacterial and antioxidant effects, reinforcing its potential within the One Health approach as a natural, sustainable product for mitigating antimicrobial resistance. Supporting future studies that evaluate their bioactivity and efficacy in vivo in veterinary and clinical settings would provide valuable information on their ecological diversity and therapeutic potential, reinforcing their role as functional products in sustainable animal and public health strategies.

## Figures and Tables

**Table 1:** MICs (µg/mL) determination and antibiotic resistance profile of antibiotics for six strains isolated from exudative skin lesions in dogs.

	*Escherichia coli*	*Enterobacter cloacae*	*Enterococcus faecium*	*Pasteurella multocida*	*Staphylococcus aureus*	*Pseudomonas aeruginosa*
Ampicilin	≥32 (R)	-	-	-	-	-
Amoxicilin/clavulanic acid	≥32 (R)	≥32 (R)	-	≥32 (R)	-	-
Cefpodoxime	≥8 (R)	≥8 (R)	-	-	-	-
Cefovecin	≥8 (R)	≥8 (R)	-	-	-	≥8 (R)
Ceftazidime	≥64 (R)	32 (R)	-	-	-	2 (R)
Ceftiofur	≥8 (R)	≥8 (R)	-	≥8 (R)	-	≥8 (S)
Imipenem	≥16 (R)	0.5 (S)	-	≥16 (R)	-	2 (S)
Ciprofloxacin	≥4 (R)	≥4 (R)	-	≥4 (R)	-	≥4 (R)
Enrofloxacin	≥4 (R)	≥4 (R)	≥4 (S)	≥4 (R)	≥4 (R)	≥4 (R)
Marbofloxacin	≥4 (R)	≥4 (R)	≥4 (R)	-	≥4 (R)	2 (R)
Pradofloxacin	-	-	-	-	≥4 (R)	-
Doxycycline	≥16 (R)	8 (R)	≥16 (R)	≥16 (R)	8 (S)	-
Chloramphenicol	16 (R)	8 (S)	≤4 (S)	-	8 (S)	-
Trimethoprim/Sulfamethoxazole	≥320 (R)	≥320 (R)	-	≥320 (R)	≥320 (R)	-
Erythromycin	-	-	≥8 (R)	-	≥8 (R)	-
Gentamicin	-	≥16 (R)	-	-	≥16 (R)	≤1 (S)
Kanamycin	-	-	-	-	≥64 (R)	-
Clindamycin	-	-	-	-	≥4 (R)	-
Tetracycline	-	-	≥16 (R)	-	≥16 (R)	-

Susceptibility classification: R: resistance; S: sensitive.

**Table 2:** Minimum inhibitory concentration (MIC) of Chilean honey extracts against multidrug-resistant (MDR) canine bacterial isolates.

MDR Strain	MIC % (*w*/*v*) by Honey Type			
	Type 1	Type 2	Type 3	Type 4
*Escherichia coli*	3.13	3.13	3.13	3.13
*Enterobacter cloacae*	12.5	12.5	12.5	12.5
*Enterococcus faecium*	3.13	3.13	3.13	3.13
*Pasteurella multocida*	12.5	12.5	12.5	12.5
*Staphylococcus aureus*	3.13	3.13	3.13	3.13
*Pseudomonas aeruginosa*	12.5	12.5	12.5	12.5
*E. coli* ATCC 25922	3.13	3.13	3.13	3.13

**Table 3:** Pairwise comparisons of antimicrobial activity among honey types obtained using Dunn’s test with Bonferroni correction.

Honey Type Comparison	*p*-Value
1–2	1.00
1–3	0.79
2–3	0.81
1–4	0.07
2–4	0.07
3–4	1.00

**Table 4:** Dose-dependent inhibition of bacterial growth by increasing honey concentrations (% *w*/*v*).

Honey Concentrations (% *w*/*v*)	Median OD *	IQR **
0.20	0.58 ^a^	0.36
0.39	0.56 ^a^	0.30
0.78	0.41 ^b^	0.25
1.56	0.29 ^c^	0.23
3.13	0.27 ^d^	0.20
6.25	0.12 ^e^	0.13
12.50	0.08 ^f^	0.05
25	0.06 ^f^	0.03
50	0.05 ^f^	0.01
100	0.05 ^f^	0.00

* Groups with different letters differ significantly according to the Steel–Dwass test (*p* < 0.05). ** Values are expressed as median (IQR).

**Table 5:** Phenolic content, flavonoids, and antioxidant capacity (FRAP) of Chilean honey samples across apiaries and seasons.

Honey Sample	Total Phenolic Content (mg GAE/100 g^−1^)	Flavonoids (mg QE/100 g^−1^)	FRAP (µmol TE/100 g^−1^)
1	171.49 ± 7.89 ^b^	40.66 ± 6.89 ^b^	7740.56 ± 452.56
2	195.58 ± 4.28 ^a^	65.46 ± 4.35 ^a^	8945.56 ± 1432.55
3	188.19 ± 7.31 ^b^	60.73 ± 10.07 ^a^	8140.56 ± 252.40
4	168.82 ± 10.04 ^b^	39.82 ± 1.84 ^b^	7646.11 ± 719.25
*p* value			
Autumn	0.054	0.024	>0.05
Summer	0.0097	0.0062	>0.05

Values are expressed as mean ± standard deviation. Different letters (a–b) within the same column indicate statistically significant differences according to Tukey’s test (*p* < 0.05). GAE: gallic acid equivalents; QE: quercetin equivalents; TE: Trolox equivalents.

## Data Availability

All data generated or analyzed during this study are included in this article. Additional information supporting the findings of this study can be obtained from the corresponding author upon reasonable request.

## Abbreviations

MIC	Minimum concentration inhibitory
*w*/*v*	Weight/volume
MDR	Multidrug-resistant
GAE	Gallic acid equivalent
QE	Quercetin equivalent
FRAP	Ferric reducing antioxidant power
UTIs	Urinary tract infections
°C	Degrees Celsius
mL	Milliliter
rpm	Revolutions per minute
µm	Micrometer
h	Hour
MHB	Mueller–Hinton
µL	Microliter
CFU	Colony-forming units
nm	Nanometer
min	Minute
mg	Milligram
TPTZ	2,4,6-Tris(2-pyridyl)-s-triazine
mM	Millimolar
NaCO_3_	Sodium Carbonate
Fe^+3^	Ferric iron
Fe^+2^	Ferrous
pH	Hydrogen potential
HCl	Hydrochloric acid
No.	Number
μmol	Micromole
OD	Optical density
ANOVA	Analysis of variance
SAS	Statistical Analysis System
IQR	Interquartile range
df	Degree freedom
g	Grams
TE	Trolox equivalent
TFC	Total flavonoid content
TPC	Total phenolic content
kg	Kilogram
